# cTnIR193H restrictive cardiomyopathy mice satisfy high-energy metabolic demands through regulating glucose metabolism

**DOI:** 10.1016/j.gendis.2025.101784

**Published:** 2025-07-25

**Authors:** Min Luo, Lingjuan Liu, Wenjing Yuan, Junjun Quan, Mi Li, Jie Tian

**Affiliations:** a Department of Cardiology, Children’s Hospital of Chongqing Medical University, Chongqing 400014, China; b National Clinical Research Center for Child Health and Disorders, Chongqing 400014, China; c Ministry of Education Key Laboratory of Child Development and Disorders, Chongqing 400014, China; d Chongqing Key Laboratory of Structural Birth Defect and Reconstruction, Chongqing 400014, China; e National Clinical Key Cardiovascular Specialty, Chongqing 400014, China; f Key Laboratory of Children’s Important Organ Development and Diseases of Chongqing Municipal Health Commission, Chongqing 400014, China

**Keywords:** cTnIR193H mutation, Glucose and fatty acid intake, Glucose metabolism, PI3K/Akt, Restrictive cardiomyopathy

## Abstract

This work aims to investigate the energy metabolism in mice with restrictive cardiomyopathy induced by cardiac troponin I (cTnI) R193H mutation. Echocardiography was used to monitor cardiac function. ATP content and ATPase activity were detected with relevant kits. The expression levels of GLUT4, FAT/CD36, and PI3K/AKT pathway proteins were detected. Proteomics and phosphorylation omics were used to analyze the differential expression and modification of cardiac proteins and related pathways, respectively. The utilization of cardiac energy substrates was investigated using relevant kits. The isovolumic relaxation time of 4-month-old cTnI193His-M mice was significantly prolonged (*P* < 0.01); Cardiac ATP content, ATPase activity, and mitochondrial number were significantly increased (*P* < 0.05, *P* < 0.01, and *P* < 0.01, respectively); GLUT4 expression level increased (*P* < 0.01); the expression level of CD36 decreased (*P* < 0.01). Proteomic results showed that the glycolytic/gluconeogenic pathway was up-regulated. Phosphorylation omics was enriched in the inositol phosphate metabolism pathway and PI3K/AKT pathway. In conclusion, at the early stage of diastolic dysfunction, cTnI193His-M mice may increase glucose uptake and metabolism through the PI3K/AKT pathway to satisfy the high energy demand, which may contribute to the development of myocardial fibrosis and heart failure.

## Introduction

Cardiomyopathy is a group of heterogeneous cardiac diseases caused by various mechanical and electrical abnormalities of the heart. Based on clinical manifestations, cardiomyopathy is primarily divided into hypertrophic cardiomyopathy, dilated cardiomyopathy, and restricted cardiomyopathy (RCM). RCM is characterized by diastolic dysfunction caused by decreased left ventricular compliance.^1^ Its clinical phenotype is more life-threatening, and its survival rate is lower than that observed in the other two cardiomyopathy categories. RCM is mainly caused by cardiac myofibroin mutations; of these, cardiac troponin I (cTnI) mutation is most common. Mutations in several sites in cTnI can cause RCM; however, the K178E and R192H mutations of cTnI gene are most harmful.^2^^,^^3^

Previously, it was believed that RCM caused by the cTnI mutation was primarily due to the mutation of the carboxy-terminal gene, which changed the calcium sensitivity of cardiac troponin, resulting in diastolic dysfunction^4^^,^^5^; cTnI mutations regulate calcium sensitivity via epigenetic inheritance.^6^ In addition, a previous study in a mouse model of cTnI knockdown-induced RCM reported significantly increased ATP content and ATPase activity in the heart, suggesting that the heart of this model is in a state of high energy metabolism.^7^ The heart is a large energy-consuming organ,^8^ and 90%–95% of its energy is supplied by mitochondria.^9^ More than 70% of the energy is produced by fatty acids, and the remaining 30% by glucose, amino acids, and acetone.^10^ Glycolysis and glucose oxidation require less oxygen (6 mol of oxygen per mole of glucose) to produce ATP compared with fatty acid oxidation (23 mol of oxygen per mole of palmitic acid), and increased glucose oxidation and decreased fatty acid metabolism increase myocardial energy efficiency by up to 30%.^11^ The activation of phosphatidylinositol 3 kinase (PI3K)-protein kinase B (Akt) signaling pathway could promote glucose uptake,^12^ and one study found that cTnI-interacting proteins were involved in the signaling of the PI3K-Akt pathway.^13^ Long-term increased glucose metabolism could increase oxidative stress through the production of reactive oxygen species in mitochondria,^14^ leading to the reduction of myocardial contractility and eventually inducing myocardial fibrosis.^15^

In conclusion, we raised the following scientific questions: whether the heart of a mouse with cTnI mutation-induced RCM is in a state of high energy metabolism, and whether the utilization of glucose and fatty acids is altered to meet their energy requirements. Therefore, the purpose of this study was to investigate the energy metabolic status of the heart and the regulation of glucose and fatty acid utilization using a murine model of RCM caused by cTnIR193H mutation.

## Material and methods

### Experimental animals

The present study was approved by the Ethical Committee on Animal Research of Chongqing Medical University. The procedures were performed based on standard animal care established by the Ethical Committee. The mice were purchased from the Animal Center of Chongqing Medical University. Since the mouse cTnI193 site is homologous to the human cTnI192 site, we expressed the mutant protein cTnI193His in transgenic mice to simulate a mutation of human cTnI C-terminal arginine 192.^16^ A cTnI193His-M mouse model was selected in this experiment,^17^ which was used as the experimental group, named the cTnI193His-M group or RCM group. Wild-type (WT) mice born in the same litter were used as the control group. Mice were housed in a specific-pathogen-free room under standard laboratory conditions at 24 ± 1 °C. A 12 h/12 h light/dark cycle (from 7:00 a.m. to 7:00 p.m.) was used in the breeding room.

### Primary cardiomyocyte culture and transfection

Extraction and culture of primary neonatal cardiomyocytes from mice were performed as previously described.^18^ Adenoviruses (AV) with mutant cTnIR193H-GFP (cTnIR193H-AV) and negative controls with GFP (NC-AV) (Shanghai Jikai Gene Technology Co., China) were transfected into the cells at a multiplicity of infection of 40.^19^ The cells were divided into the control group, NC-AV group, cTnIR193H-AV group, and cTnIR193H-AV plus LY294002 (MedChemExpress, HY-10108, China; 20 μM, added immediately after starvation transfection of the virus for 12 h) group.

### Echocardiography

Continuous cardiac ultrasound monitoring was performed in 2-, 3-, and 4-month-old mice. Mice were anesthetized with isoflurane and fixed on a hard plate in the supine position. Anesthesia was maintained using isoflurane through a mask, precardiac hair was removed, and the ultrasonic coupling agent was applied. The cardiac function and ventricular wall of the mice were detected in the parasternal short-axis view and apical four-chamber view of the heart using an ultrasound machine (VEVO2100, FUJIFILM VisualSonics, Canada; probe frequency 30 Hz).

### ATP concentration and ATPase activity measurement

After cardiac ultrasound showed diastolic dysfunction, the mice were sacrificed (cervical dislocation), and their hearts were removed. Then, the ATP concentration and ATPase activity in mouse heart muscle were detected using the ATP Assay Kit (Beyotime, S0026, China) and the ATPase Assay Kit (Nanjing Jiancheng Bioengineering Institute, A070-1-1, China), according to the manufacturer’s instructions. Approximately 72 h after virus transfection, primary cardiomyocytes were collected for ATP concentration detection using the ATP Assay Kit (Beyotime, S0026, China).

### Transmission electron microscopy

The mouse left ventricle was cut into 1 cubic millimeter pieces. The tissue was prefixed with 3% glutaraldehyde and then fixed using 1% osmium tetroxide, dehydrated in a series of acetone, infiltrated with Epox 812 for a long period, and embedded. The semi-thin sections were stained with methylene blue, and ultra-thin sections were cut with a diamond knife and stained with uranyl acetate and lead citrate. Sections were examined with a JEM-1400-FLASH transmission electron microscope.

### Real-time quantitative PCR

Total RNA was extracted from heart tissue using the Simply P Total RNA Extraction Kit (BioFlux, BSC52S1, China). Thereafter, extracted RNA was reverse-transcribed into cDNA using the PrimeScript™ RT Reagent Kit with gDNA Eraser (Perfect Real Time) (TaKaRa, RR047A, Japan). Real-time quantitative PCR was performed using the CFX96 Real-Time PCR System (Bio-RAD, the United States) and the QuantiFast SYBR® Green RT-PCR Kit (QIAGEN, No. 169016155, Germany). The sequences of mouse-specific primers used were 5′-TGGAAGGAAAAGGGCC ATGCTG-3′ (forward) and 5′-CAATGAGGAATCGTCCAAGGATG-3′ (reverse) for glucose transporter 4 (GLUT4), and 5′-TGGCCTTACTTGGGATTGG-3′ (forward) and 5′-CC AGTGTATATGTAGGCTCA-3′ (reverse) for CD36/fatty acid translocase (FAT).

### Immunohistochemical staining

Following removal, the heart was rinsed in phosphate-buffered saline and subsequently placed in 4% paraformaldehyde for 48 h. Subsequently, it was dehydrated and embedded in paraffin and cut into 5-μm-thick slices. Sections were dewaxed, treated with antigen repair and 3% hydrogen peroxide, and subsequently sealed with serum. The sections were incubated with primary antibodies (GLUT4, 1:100, Wanleibio, WL02425, China; CD36, 1:100, Wanleibio, WL02390, China) at 4 °C for 14 h. Next, the sections were incubated with the secondary antibody (horseradish peroxidase-labeled) at 25 °C for 50 min, followed by 3,3′-diaminobenzidine chromogenic staining and hematoxylin staining, dehydration, and sealing. Finally, the stained sections were observed and photographed under a microscope (Nikon, Japan). The results were analyzed using Image-Pro Plus 6.0.

### Western blotting analysis

Proteins were extracted from mouse heart tissue using RIPA Lysis Buffer (Beyotime, P0013B, China). Western blotting analysis was used to detect the expression levels of GLUT4, CD36, PI3K, Akt, and p-Akt using specific antibodies (GLUT4, 1:2000, Proteintech, 66846-1-Ig, China; CD36, 1:500, Wanleibio, WL02390, China; PI3K, 1:1000, Wanleibio, WL03380, China; Akt, 1:500, Wanleibio, WL0003b, China; p-Akt, 1:500, Wanleibio, WLP001a, China). Glyceraldehyde 3-phosphate dehydrogenase (GAPDH) (1:2000, ORIGENE, F004, United States) was used as an internal reference. Image Lab software was used to analyze the gray values of bands obtained and calculate the relative expression levels of related proteins.

### Immunofluorescence

Mice hearts were rinsed in phosphate-buffered saline and subsequently placed in 4% paraformaldehyde for 24 h at 25 °C. Following dehydration with sucrose, hearts were embedded in an optimal cutting temperature compound and cut into 8-μm slices. After washing with phosphate-buffered saline, slices were sealed with 5% bovine serum albumin at 25 °C for 30 min and incubated with primary antibodies (GLUT4, 1:50, Wanleibio, WL02425, China; CD36, 1:50, Wanleibio, WL02390, China) at 4 °C for 14 h. The sections were incubated with the secondary antibody (rhodamine (TRITC)-conjugated AffiniPure goat anti-rabbit IgG(H + L), 1:200, ZSGB-BIO, ZF-0316, China) at 25 °C for 1 h. Following staining with 4′,6-diamidino-2-phenylindole (DAPI) (Beyotime, C1006, China), the slices were sealed and photographed using a confocal laser microscope (Nikon, Japan). NIS-Elements Analyze software was used to analyze the fluorescence intensity.

### Proteomics and phosphorylation omics

Proteomics and phosphorylation omics were based on liquid chromatography with tandem mass spectrometry analyses. These analyses were performed as previously described.^20^ Briefly, proteins were extracted from the heart tissues of cTnI193His-M mice and WT mice. Thereafter, they were analyzed using liquid chromatography with tandem mass spectrometry with the help of Shanghai Applied Protein Technology Co., Ltd., China. Mass spectrometry data were combined and searched using the MaxQuant 1.5.3.17 software for identification and quantification. Fold change >2 or <0.5 and *P* < 0.05 were used as thresholds to screen differentially expressed proteins. Following annotation steps, Blast2GO was subjected to gene ontology (GO) annotation on the target protein collection. The studied proteins were blasted against the online Kyoto Encyclopedia of Genes and Genomes (KEGG) database to retrieve their KEGG orthology identifications and were subsequently mapped to KEGG pathways.

### Glucose and lactic acid measurement

Following the removal of mouse heart tissue, glucose and lactic acid concentrations were detected using the Glucose Content Assay Kit (Solarbio, BC2500, China) and Lactic Acid Content Assay Kit (Solarbio, BC2235, China). Cell culture media were collected at 72 h, 96 h, and 108 h after transfection, and glucose concentration in the culture media was detected using a glucose content assay kit (Beyotime, S0201S, China). All steps were performed in strict accordance with the manufacturer’s instructions.

### Measurement of oxidative stress-related indicators

The primary cardiomyocytes were transfected with virus after 130 h; the culture medium was removed, and then the cells were washed with phosphate-buffered saline twice. Malondialdehyde content and superoxide dismutase activity were detected using a lipid peroxidation malondialdehyde assay kit (Beyotime, S0131s, China) and a total superoxide dismutase assay kit with WST-8 (Beyotime, S0101s, China) to evaluate the oxidative stress of the cells. All steps were performed in strict accordance with the manufacturer’s instructions.

### Statistical analysis

All results were presented as mean ± standard deviation. Comparisons between the two groups were performed using a *t*-test. The Mann–Whitney test was used to analyze data that did not show normal distribution. Statistical significance was set at *P* < 0.05. All statistical analyses were performed using GraphPad Prism (version 8.3.0) software.

## Results

### Cardiac function

**Figure 1:**
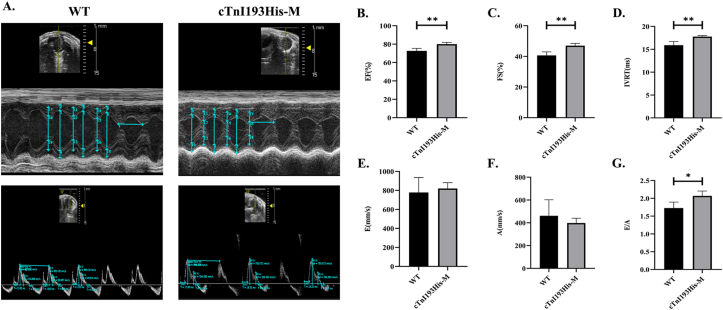
Cardiac function of 4-month-old mice. **(A)** Representative M-mode echocardiography of short axis and representative PW-mode echocardiography of four-chamber section. **(B)** Left ventricular ejection fraction (EF%). **(C)** Left ventricular fractional shortening (FS%). **(D)** Left ventricular isovolumic relaxation time (IVRT). **(E)** E peak. **(F)** A peak. **(G)** E/A. *n* = 4; ∗*P* < 0.05; ∗∗*P* < 0.01.

Ultrasound monitoring revealed no significant difference in diastolic and contractile functions between the cTnI193His-M and WT groups (2- and 3-month-old mice). However, the isovolumic relaxation time and E/A ratio (the peak early-diastolic (E) and peak end-diastolic (A) transmission velocity) were significantly increased (*P* < 0.01 and *P* < 0.05, respectively) in 4-month-old mice of the cTnI193His-M group, indicating diastolic dysfunction at this stage; ejection fraction and fractional shortening were also significantly increased (*P* < 0.05) (Fig. 1A–G; Table 1).

**Table 1:** Echocardiographic data.

	2M		3M		4M	
	WT	cTnI193His-M	WT	cTnI193His-M	WT	cTnI193His-M
Heart rate (times/min)	459 ± 10	483 ± 28	480 ± 33	456 ± 14	479 ± 26	496 ± 11
IVS; d (mm)	0.43 ± 0.05	0.49 ± 0.06	0.43 ± 0.05	0.51 ± 0.06	0.62 ± 0.09	0.56 ± 0.06
IVS; s (mm)	0.67 ± 0.06	0.79 ± 0.09	0.59 ± 0.13	0.66 ± 0.04	0.89 ± 0.11	0.87 ± 0.05
LVID; d (mm)	3.14 ± 0.26	3.18 ± 0.36	3.36 ± 0.34	3.31 ± 0.15	3.25 ± 0.21	2.88 ± 0.48
LVID; s (mm)	1.89 ± 0.50	1.66 ± 0.25	2.36 ± 0.65	2.18 ± 0.18	1.93 ± 0.16	1.53 ± 0.29
LVPW; d (mm)	0.65 ± 0.10	0.62 ± 0.07	0.58 ± 0.05	0.62 ± 0.13	0.72 ± 0.11	0.65 ± 0.07
LVPW; s (mm)	1.04 ± 0.19	1.04 ± 0.06	0.80 ± 0.07	0.86 ± 0.05	0.93 ± 0.09	0.89 ± 0.14
LV Vol; d (μL)	39.31 ± 7.68	40.92 ± 11.65	46.74 ± 11.63	44.66 ± 4.81	42.75 ± 6.72	32.84 ± 14.31
LV Vol; s (μL)	12.09 ± 7.60	8.19 ± 3.07	21.11 ± 13.59	15.88 ± 3.33	11.72 ± 2.40	6.74 ± 3.58
EF (%)	70.78 ± 14.68	80.30 ± 3.88	57.52 ± 18.53	63.96 ± 10.15	72.69 ± 2.79	80.02 ± 1.84^a^
FS (%)	40.31 ± 12.32	47.80 ± 3.87	30.49 ± 12.91	34.21 ± 7.21	40.68 ± 2.30	47.05 ± 1.43^a^
IVCT (ms)	8.35 ± 0.81	8.75 ± 0.83	10.09 ± 2.16	7.59 ± 0.58	8.68 ± 1.12	8.06 ± 0.94
IVRT (ms)	18.52 ± 1.93	16.60 ± 2.21	18.89 ± 0.28	20.93 ± 2.39	15.90 ± 0.80	17.78 ± 0.23^b^
A (mm/s)	438.06 ± 83.16	338.54 ± 72.18	506.47 ± 68.38	376.46 ± 90.16	461.36 ± 141.00	398.28 ± 42.03
E (mm/s)	615.86 ± 53.07	666.68 ± 164.38	613.03 ± 81.50	556.10 ± 79.94	778.61 ± 157.72	820.65 ± 61.63
E/A	1.44 ± 0.30	2.01 ± 0.45	1.21 ± 0.01	1.58 ± 0.62	1.72 ± 0.17	2.07 ± 0.13^a^

Note: IVS; d, interventricular septum thickness at end-diastole; IVS; s, interventricular septal thickness at end systolic; LVID; d, left ventricular internal dimension at end-diastole; LVID; s, left ventricular internal dimension at end-systole; LVPW; d, left ventricular posterior wall thickness at end-diastole; LVPW; s, left ventricular posterior wall thickness at end-systole; LV Vol; d, left ventricular volume at end-diastole; LV Vol; s, left ventricular volume at end-systole; EF, ejection fraction; FS, fractional shortening; IVCT, isovolumic contraction time; IVRT, isovolumic relaxation time; A, A peak; E, E peak. *n* = 4.

a *P* < 0.05 versus the WT group at the same age.

b *P* < 0.01 versus the WT group at the same age.

### Cardiac ATP concentration, ATPase activity, and mitochondrial quantity

**Figure 2:**
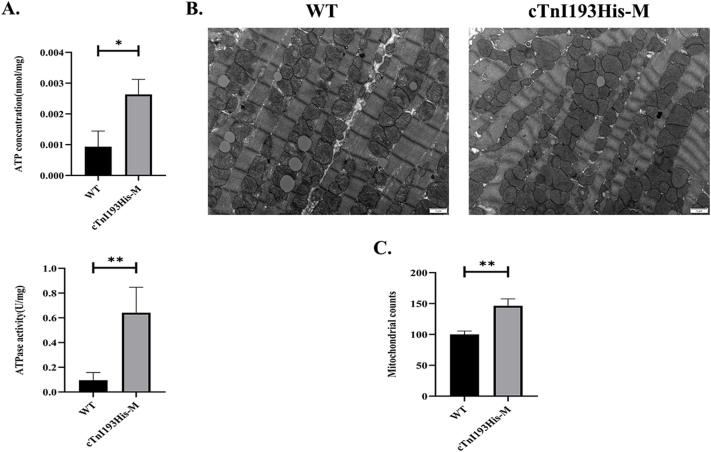
Cardiac energy metabolism. **(A)** ATP concentration (*n* = 3) and ATPase activity (*n* = 4) in the hearts of 4-month-old WT and cTnI193His-M mice. **(B)** Electron micrographs of sections from 4-month-old WT and cTnI193His-M mouse hearts (*n* = 3; 12,000×). **(C)** Statistical analysis of mitochondrial number under an electron microscope. ∗*P* < 0.05; ∗∗*P* < 0.01.

Our results showed that myocardial ATP concentration and ATPase activity significantly increased in 4-month-old cTnI193His-M mice (*P* < 0.05 and *P* < 0.01, respectively) (Fig. 2A), but showed no significant difference in 3-month-old cTnI193His-M mice (Fig. S1). Transmission electron microscopy showed no significant difference in mitochondrial structure between the two groups. No swelling, ridge fracture, and autophagy were found in the mitochondria (Fig. 2B). However, the number of mitochondria in 4-month-old cTnI193His-M mice was significantly increased when compared with that in 4-month-old WT mice (*P* < 0.01) (Fig. 2C).

### Cardiac GLUT4 and CD36 mRNA and protein expression levels

**Figure 3:**
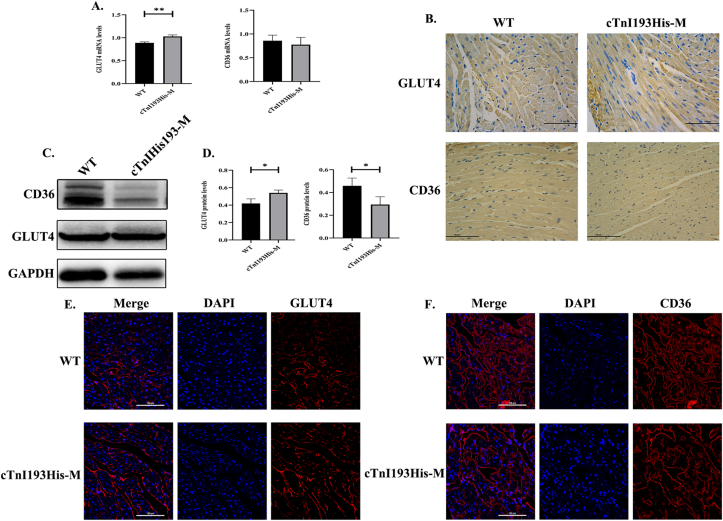
GLUT4 and CD36 expression levels. **(A)** GLUT4 and CD36 mRNA expression levels (WT, *n* = 4; cTnI193His-M, *n* = 3). **(B)** Immunohistochemical staining of GLUT4 and CD36 in heart tissue (*n* = 3). Photographs were taken at 400× using an electron microscope. **(C)** CD36 and GLUT4 protein levels (*n* = 3). **(D)** Statistical analysis of western blotting results for the detection of protein expression. **(E)** GLUT4 immunofluorescence staining on the cell membrane (*n* = 3). **(F)** CD36 immunofluorescence staining on the cell membrane (*n* = 3). Photographs were captured at 400× using a confocal laser. ∗*P* < 0.05; ∗∗*P* < 0.01.

Quantitative PCR showed that the expression level of GLUT4 mRNA in the cTnI193His-M group was significantly high (*P* < 0.01), while the expression level of CD36 mRNA had a downward trend with no statistical significance (Fig. 3A). Immunohistochemistry showed that the level of GLUT4 protein in the cTnI193His-M group was high (*P* < 0.01), CD36 protein expression was low (*P* < 0.01) (Fig. 3B; statistical charts are provided in Fig. S2), which was further verified using western blotting analysis (*P* < 0.05) (Fig. 3C, D). The immunofluorescence assay was used to detect the relative expression levels of GLUT4 and CD36 on the myocardial cell membrane; the results showed that the GLUT4 expression level in the cTnI193His-M group was significantly high (*P* < 0.05), while the expression level of CD36 showed a downward trend with no statistical significance (Fig. 3E, F; statistical charts are provided in Fig. S3).

### Proteomic analysis and glucose metabolism in the heart

**Figure 4:**
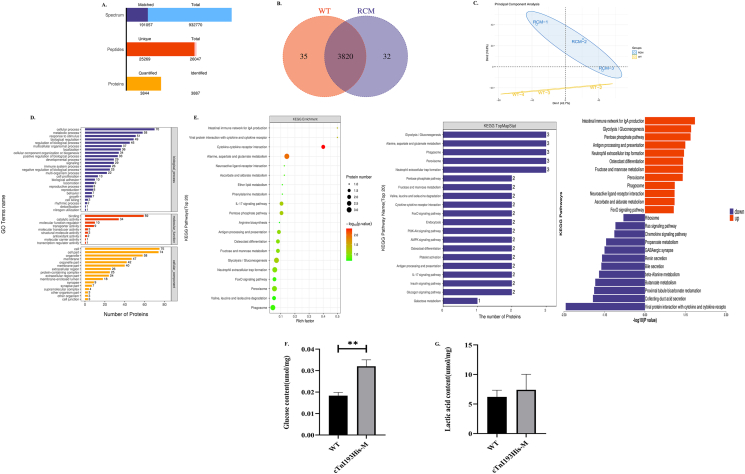
Proteomic analysis and glucose metabolism in the heart. **(A)** Proteomics protein identification and quantitative results statistics. **(B)** A Venn diagram showed the overlapping differentially expressed proteins between the RCM group and the WT group. **(C)** The principal component analysis of all samples. **(D)** GO annotation statistics of differentially expressed proteins in the RCM_vs_WT group. The ordinate represented the secondary GO function annotation information, including biological processes, molecular functions, and cell components, which were successively distinguished by blue, red, and orange. The abscissa indicated the number of differentially expressed proteins in each functional category. **(E)** KEGG enrichment analysis, top20 KEGG TopMapStat sorted by the number of proteins enriched on the KEGG pathway, and up-down-regulation analysis of KEGG enrichment pathway. The blue represents down-regulation and the red represents up-regulation. **(F)** Glucose content in heart tissue (*n* = 3). **(G)** Lactic acid content in heart tissue (WT, *n* = 5; cTnI193His-M, *n* = 4). ∗∗*P* < 0.01.

Proteins extracted from the left ventricle were subjected to label-free quantitative proteomics. A total of 932,770 spectra with 26,047 matching known peptides were obtained and 3887 proteins were identified (Fig. 4A). Venn diagram was used to analyze the overlap of protein identification between different groups (Fig. 4B). The principal component analysis results showed completely different patterns between the two groups (Fig. 4C). The results of GO level2 showed that most differentially expressed proteins were mainly included in cellular process and metabolic process in biological process (Fig. 4D). Enrichment analysis of the KEGG pathway revealed that the most observably affected pathways included cytokine–cytokine receptor interaction, alanine, aspartate and glutamate metabolism, and glycolysis/gluconeogenesis. Top20 KEGG TopMapStat sorted by the number of proteins enriched in the KEGG pathway revealed that glycolysis/gluconeogenesis was at the top. The glycolysis/gluconeogenesis pathway was up-regulated (Fig. 4E). Further, detection of glucose and lactic acid concentration in heart tissue showed that the glucose concentration in the cTnI193His-M group was significantly high (*P* < 0.01) (Fig. 4F); however, there was no significant difference in lactic acid concentration between the two groups (Fig. 4G).

### Phosphorylation omics analysis and PI3K/AKT pathway-related protein expression in the heart

**Figure 5:**
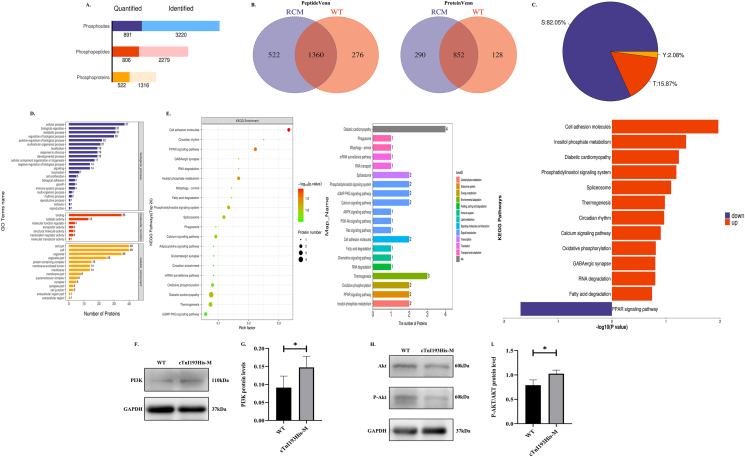
Phosphorylation omics analysis and expression of PI3K/AKT pathway-related proteins in the heart. **(A)** Phosphorylation omics identification and quantitative results statistics. **(B)** Venn diagram of phosphopeptides identified between all sample groups and Venn diagram of phosphoproteins identified between all sample groups. **(C)** Distribution ratio diagram of S/T/Y phosphorylation modification sites. **(D)** GO annotation statistics of differentially expressed phosphoproteins corresponding to differentially expressed phosphopeptides in the RCM and WT groups. The ordinate represents the secondary GO function annotation information, including biological processes, molecular functions, and cell components, which were successively distinguished by blue, red, and orange. The abscissa represents the number of differentially expressed proteins in each functional category. **(E)** KEGG enrichment analysis, top20 KEGG TopMapStat sorted by the number of proteins enriched on the KEGG pathway, and up-down-regulation analysis of KEGG enrichment pathway. The blue represents down-regulation and the red represents up-regulation. **(F)** PI3K protein levels. **(G)** Statistical chart of PI3K protein expression levels (*n* = 3). **(H)** AKT and p-AKT protein levels. **(I)** Statistical chart of p-AKT/Akt protein expression ratio (*n* = 3). ∗*P* < 0.05.

Proteins extracted from the left ventricle were subjected to label-free quantitative phosphorylation omics. A total of 3220 phosphosites with 2279 matching known phosphopeptides were obtained and 1316 phosphoproteins were identified (Fig. 5A). Venn diagram was used to analyze the overlap of phosphopeptide and phosphoprotein identification between different groups (Fig. 5B). Pie chart was used to show the distribution of phosphorylation on different amino acids, mainly showing the distribution proportion of sites of phosphorylation on serine (S), threonine (T), and tyrosine (Y) (Fig. 5C). The results of GO level2 showed that most differentially expressed phosphoproteins were mainly included in cellular process, biological regulation, and metabolic process in biological process (Fig. 5D). Enrichment analysis of the KEGG pathway revealed that the most observably affected pathways included cell adhesion molecules, PPAR signaling pathway, and inositol phosphate metabolism. Top20 KEGG TopMapStat_level2 sorted by the number of proteins enriched in the KEGG pathway revealed that inositol phosphate metabolism and PI3K/Akt signaling pathway were found to be at the front. The inositol phosphate metabolism pathway was up-regulated (Fig. 5E).

The protein expression level of the PI3K/AKT pathway was detected by western blotting, and it was found that the PI3K protein expression level of cTnI193His-M mice was significantly increased when compared with WT mice (*P* < 0.05) (Fig. 5F, G). The p-Akt/AKT expression ratio was significantly increased in cTnI193His-M mice (*P* < 0.05) (Fig. 5H, I).

### Cellular ATP concentration, glucose metabolism, and oxidative stress

**Figure 6:**
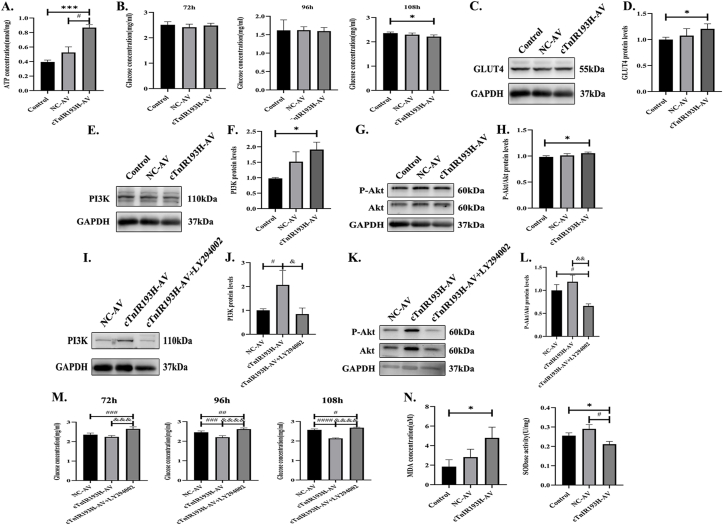
Cellular ATP concentration, glucose metabolism, and oxidative stress. **(A)** ATP concentration of primary cardiomyocytes 72 h after transfection of virus (*n* = 3). **(B)** Glucose concentration in cell culture medium, 72 (Control, NC-AV, *n* = 4, cTnIR193H-AV, *n* = 3), 96 (*n* = 5), and 108 (*n* = 4) h after transfection of virus. **(C)** GLUT4 protein levels. **(D)** Statistical chart of GLUT4 protein expression levels (*n* = 4). **(E)** PI3K protein levels. **(F)** Statistical chart of PI3K protein expression levels (*n* = 5). **(G)** AKT and p-AKT protein levels. **(H)** Statistical chart of p-AKT/Akt protein expression ratio (*n* = 4). **(I)** PI3K protein levels. **(J)** Statistical chart of PI3K protein expression levels (*n* = 5). **(K)** AKT and p-AKT protein levels. **(L)** Statistical chart of p-AKT/Akt protein expression ratio (*n* = 4). **(M)** Glucose concentration in cell culture medium, 72 (*n* = 6), 96 (*n* = 6), and 108 (*n* = 5) h after transfection of virus. **(N)** Malondialdehyde (MDA) concentration (*n* = 4) and superoxide dismutase (SOD) activity (*n* = 3) in primary cardiomyocytes 130 h after transfection of virus. ∗*P* < 0.05 versus control; ∗∗∗*P* < 0.01 versus control; ^#^*P* < 0.05 versus NC-AV; ^##^*P* < 0.01, ^###^*P* < 0.01, and ^####^*P* < 0.01 versus NC-AV; ^&^*P* < 0.05 versus cTnIR193H-AV; ^&&^*P* < 0.01, ^&&&^*P* < 0.01, and ^&&&&^*P* < 0.01 versus cTnIR193H-AV.

We found that 72 h after transfection with virus, ATP concentration of primary cardiomyocytes in cTnIR193H-AV group was significantly higher than in control group (*P* < 0.01) and NC-AV group (*P* < 0.05) *in vitro* (Fig. 6A). There was no significant difference in glucose concentration in the cell culture medium at 72 and 96 h, while at 108 h, glucose concentration in cTnIR193H-AV group was significantly lower than that in the control group (*P* < 0.05) (Fig. 6B). GLUT4 protein expression in cTnIR193H-AV group was significantly higher than that in control group (*P* < 0.05) (Fig. 6C, D). Further detection showed that PI3K protein expression level and p-Akt/Akt expression ratio were also significantly increased (*P* < 0.05) (Fig. 6E–H). After the application of the PI3K inhibitor LY294002, we found that PI3K protein expression level and p-Akt/Akt expression ratio in the cTnIR193H-AV plus LY294002 group decreased significantly compared with the cTnIR193H-AV group (*P* < 0.05 and *P* < 0.01, respectively) (Fig. 6I–L). Moreover, the glucose concentration in the cell culture medium in the cTnIR193H-AV plus LY294002 group was significantly higher than that in the cTnIR193H-AV group at 72 h, 96 h, and 108 h after viral transfection (*P* < 0.01) (Fig. 6M).

We further detected the late oxidative stress of cells, 130 h after transfection of virus showed that malondialdehyde content in cTnIR193H-AV group was significantly higher than that in the control group (*P* < 0.05); the superoxide dismutase activity in cTnIR193H-AV group was significantly lower than that in the control group and NC-AV group (*P* < 0.05) (Fig. 6N).

## Discussion

The onset and severity of RCM in a mouse model with cTnIR193H mutation were dose-dependent on mutant protein content.^17^ In this study, we chose cTnI193His-M, a mouse model with a moderate-dose cTnI mutation, as the research object. Echocardiography was conducted at 2, 3, and 4 months of age to monitor changes in cardiac function. The results showed that the isovolumic relaxation time of 4-month-old cTnI193His-M mice was significantly longer than that of 4-month-old WT mice, indicating that the impairment of diastolic function of cTnI193His-M mice began at this time.

Previously, studies on cTnI-knockout mice showed that the number of cardiac mitochondria and cardiac mitochondrial ATPase activity and ATP content were significantly increased (*P* < 0.05).^7^ In this study, we found that cardiac ATP content, ATPase activity, and the number of mitochondria were significantly increased in 4-month-old cTnI193His-M mice (*P* < 0.05, *P* < 0.01, and *P* < 0.01, respectively), indicating that high energy metabolism occurred in the heart of cTnI193His-M mice and demonstrating that high ATP production was necessary to meet the elevated energy consumption required to maintain continuous myocardial contraction. A significant increase in ATP content was also found in primary cardiomyocytes transfected with cTnIR193H-AV (*P* < 0.01 versus the control group; *P* < 0.05 versus the NC-AV group).

The energy generation of the heart can come from the utilization of various energy substrates, primarily relying on fatty acids under normal circumstances, followed by glucose.^21^ Energy substrates entering cardiac cardiomyocytes are utilized depending on the transport of related vectors. Cardiac cells primarily metabolize long-chain fatty acids,^22^ and the transport of fatty acids into cells mainly depends on CD36 found on the cell membrane.^23^ Glucose transport into cells depends on the glucose transporter receptor family, and GLUT4 is the main receptor in cardiomyocytes, followed by glucose transporter 1.^24^ We examined the transporters associated with glucose and fatty acid transport in the heart of cTnI193His-M mice and found that mRNA expression of GLUT4 was significantly elevated (*P* < 0.05), while that of CD36 was not. The discrepancy between the mRNA and protein expression levels of CD36 may be attributed to post-transcriptional regulation and post-translational modifications. Further, protein level detection showed that GLUT4 increased significantly (*P* < 0.05), while CD36 significantly decreased (*P* < 0.05). These transporters perform their transport function only when they bind to cell membranes.^25^^,^^26^ Immunofluorescence was used to detect GLUT4 and CD36 located on the cell membrane. The results showed that the relative expression level of GLUT4 in cTnI193His-M mice was high (*P* < 0.05), and the expression level of CD36 showed a downward trend. GLUT4 is an insulin-sensitive transporter and results in a sharp increase in cardiac glucose uptake after insulin stimulation or a sudden increase in cardiac activity.^27^ These results indicated that the utilization of energy substrates in cTnI193His-M mice changed after the induction of diastolic dysfunction, increasing the uptake of glucose. We examined the medium of primary cardiomyocytes transfected with cTnIR193H-AV and found a significant decrease in glucose concentration (*P* < 0.05 versus the control group), suggesting increased uptake. We also found that GLUT4 protein expression level was significantly higher in the cTnIR913H-AV group than in the control group (*P* < 0.05 versus the control group).

We used proteomics to analyze the expression of cardiac proteins in cTnI193His-M mice and WT mice. GO analysis of the obtained differential proteins showed that most of the differential proteins participated in the metabolic process. Further, enrichment by KEGG pathway analysis showed that the glycolysis/gluconeogenesis pathway was up-regulated. Then, we found that the cardiac glucose content of the cTnI193His-M mice group significantly increased (*P* < 0.01). This appeared to be caused by increased intake and up-regulation of gluconeogenesis. As glucose enters the cell or gluconeogenesis increases the intracellular glucose, ATP is generated via two main pathways, glucose oxidation and glycolysis. Since glucose oxidation and glycolysis share the same initiation pathway, both have a common starting point, from glucose to pyruvate production.^28^ We discussed whether glucose was primarily involved in glycolysis or glucose oxidation in cTnI193His-M mice. Thus, we detected lactic acid concentration in the heart, and the results showed no significant difference between the two groups, suggesting that increased glucose levels were still primarily involved in glucose oxidation.

To further explore the mechanism of increased glucose uptake, phosphorylomic tests were performed. The GO analysis of the obtained differential phosphoproteins showed that most of the differential phosphoproteins were involved in metabolic processes. Further enrichment analysis of KEGG pathway showed that the inositol phosphate metabolism pathway was up-regulated, and the PI3K/Akt pathway was also included in the Top20 KEGG TopMapStat_level2. The PI3K/AKT signaling pathway is involved in many biological processes. In glucose metabolism, PI3K activates AKT by phosphorylation, and activated AKT migrates to GLUT4 by phosphorylation of AKT160ku substrate AS160, thereby increasing glucose uptake.^29^ In our study, we found that the expression level of PI3K (*P* < 0.05) and the proportion of activated AKT (*P* < 0.05) in cTnI193His-M mouse heart and cTnIR913H-AV cells were significantly increased; and these (*P* < 0.05 and *P* < 0.01, respectively) in the cTnIR193H-AV plus LY294002 group decreased significantly compared with the cTnIR193H-AV group, accompanied by a significant reduction in glucose uptake (*P* < 0.01). This suggests that the cTnI mutation may regulate glucose uptake through the PI3K/AKT pathway.

Many studies have linked the relationship between inositol and metabolism. For example, inositol isomers like myoIns and d-chiro-inositol have been shown to reduce the risk of metabolic diseases, including diabetes and dyslipidemia.^30^^,^^31^ In addition, d-chiro-inositol increased the protein expression of insulin receptor substrate 2 (IRS2), PI3K, Akt, GLUT4, and p-Akt in hepatocytes.^32^ This finding might give a mechanism of how d-chiro-inositol regulates glucose metabolism by up-regulating the insulin receptor, GLUT4, and PI3K-Akt cascade. In this study, the inositol phosphate metabolism pathway was up-regulated, and enrichment of the PI3K/Akt pathway was found through phosphoproteomics. The expression of PI3KAP1 in cTnIR193H mice was significantly higher than that in WT mice.^33^ Moreover, proteins interacting with cTnI were involved in the signal transduction of the PI3K/Akt pathway, mainly integrin protein families (such as Itga5, Itga6, Itgav, and Itgab1).^13^ When integrins were stimulated by signals such as extracellular mechanical forces, they could recruit a cytoplasmic tyrosine kinase to further activate PI3K/Akt and exert biological effects.^34^^,^^35^ This further suggests that the PI3K/Akt pathway might play an important role in the glucose metabolism reprogramming of RCM caused by cTnIR193H mutation, and they might be interrelated through the integrin protein family.

Long-term dominance of glucose metabolism has several side effects, such as oxidative stress. Oxidative stress can accelerate myocardial cell apoptosis and cellular DNA damage.^36^ We used primary cardiomyocytes transfected with cTnIR193H-AV to monitor oxidative stress in the later stage. The results showed that the content of malondialdehyde, the end-product of lipid oxidation, was significantly increased when compared with the control group (*P* < 0.05), while the content of superoxide dismutase was significantly decreased when compared with the other two groups (*P* < 0.05), suggesting that cardiomyocytes were in a peroxidation state in the later stage.

In the development of heart failure, insufficient energy supply plays an important role.^37^ We know that 1 mol of glucose oxidation can provide 30/32 mol of ATPs, while 1 mol of palmitic acid oxidation can provide 106 mol of ATPs; thus, fatty acid oxidation provides more energy than glucose oxidation. Other studies have found that maintaining a high level of fatty acid oxidation and preventing metabolic conversion to increased glucose oxidation and glycolysis can maintain myocardial function under stress overload.^38^ Available data suggests that high fatty acid utilization may actually be beneficial in the development of heart failure, which is similar to the “obesity paradox”.

However, our current study was limited to the phenomenon of energy metabolism and utilization of glucose and fatty acids in the early stage of cardiac diastolic dysfunction in RCM caused by the cTnIR193H mutation. In future studies, we plan to investigate cardiac glucose and lipid metabolism in model mice before the onset of diastolic dysfunction, with the aim of identifying the key transition points between lipid and glucose metabolism. In addition, we plan to pre-intervene in the PI3K/Akt signaling pathway in the cTnI193His-M model and observe the changes in glucose and lipid metabolism and the incidence of RCM, with the aim of elucidating the role of these alterations in cTnIR193H mutation-induced RCM.

In summary, at the early stage of diastolic dysfunction in cTnI193His-M mice, cardiometabolic transformation occurs, which may increase the dependence on glucose oxidation to meet higher energy requirements through the PI3K/AKT signaling pathway. Long-term glucose metabolism may lead to side effects and insufficient energy supply, thereby damaging the heart and promoting the development of the disease. This may be a pathogenesis of late myocardial fibrosis and heart failure in cTnI R193H mutation-induced RCM mice. It may provide a new treatment idea for preventing the occurrence of advanced heart failure.

## CRediT authorship contribution statement

**Min Luo:** Validation, Data curation, Methodology, Formal analysis, Visualization, Investigation, Conceptualization, Writing – original draft, Resources. **Lingjuan Liu:** Methodology, Data curation, Visualization, Formal analysis, Validation, Investigation, Conceptualization, Writing – original draft, Resources. **Wenjing Yuan:** Formal analysis, Investigation. **Junjun Quan:** Writing – review & editing, Conceptualization, Supervision, Methodology. **Mi Li:** Writing – review & editing, Conceptualization. **Jie Tian:** Methodology, Conceptualization, Writing – review & editing, Funding acquisition, Supervision.

## Ethics declaration

The present study was approved by the Ethical Committee on Animal Research of Chongqing Medical University (License number: SYXK YU 2012-0001). The procedures were performed based on standard animal care established by the Ethical Committee.

## Funding

This study was supported by a grant from the 10.13039/501100001809National Natural Science Foundation of China (No. 81974030).

## Conflict of interests

Jie Tian is the Associate Editor of *Genes & Diseases*, but he/she has no involvement in the peer-review of this article and has no access to information regarding its peer-review. The rest authors have no competing interests to declare.
