# Supplementary material for: cTnIR193H restrictive cardiomyopathy mice satisfy high-energy metabolic demands through regulating glucose metabolism
Source: Genes Dis. 2025 Jul 25;13(2):101784. doi: 10.1016/j.gendis.2025.101784 (PMC12723128; doi:10.1016/j.gendis.2025.101784)
Supplement: Multimedia component 1 [file mmc1.docx]

**Fig.1**

**
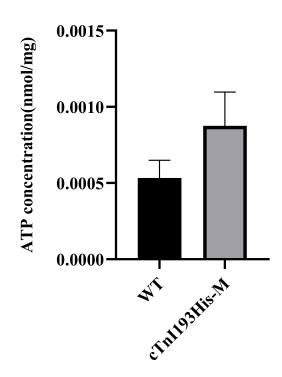

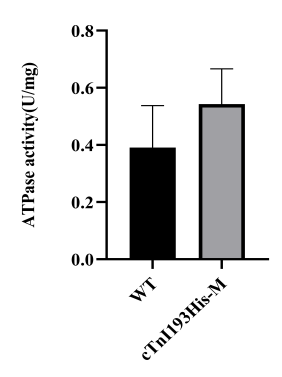
**

**Fig.1 Cardiac ATP content and ATPase activity.** ATP concentration (WT--n=3; cTnI193His-M--n=4) and ATPase activity (WT--n=4; cTnI193His-M--n=3) in the heart of 3-month-old mice.

**Fig.2**


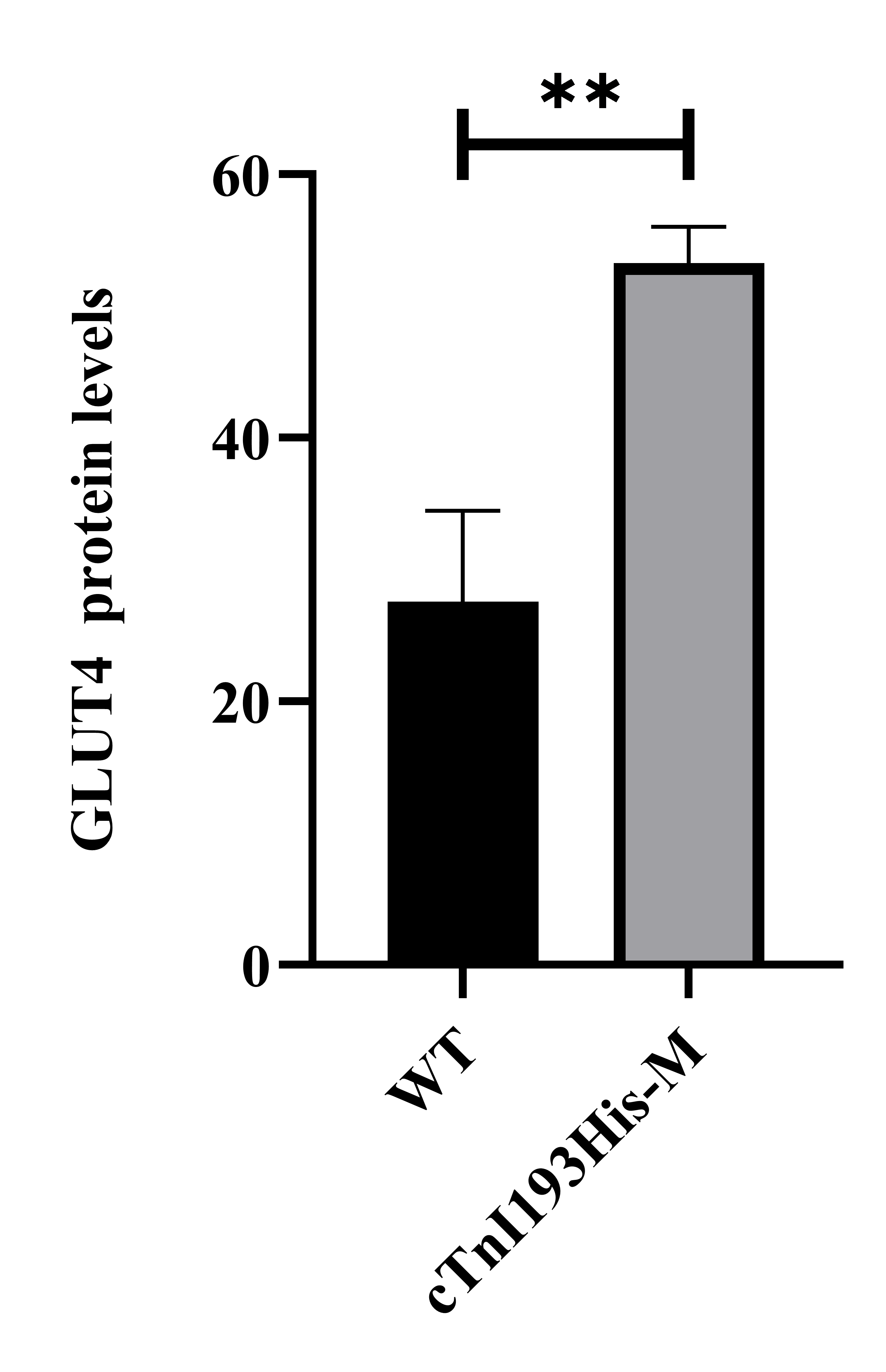

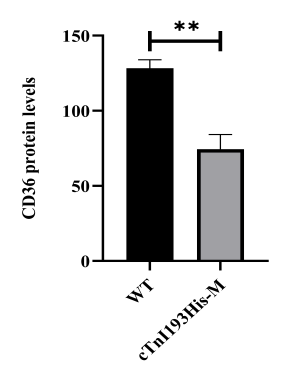


**Fig.2 Immunohistochemical expression levels of GLUT4 and CD36.** n=3. ** represents *p* < 0.01.

**Fig.3**

**
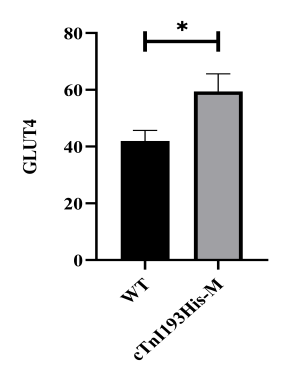

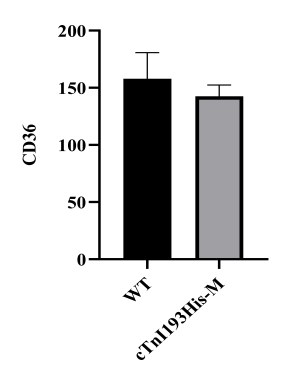
**

**Fig.3 Immunofluorescence expression levels of GLUT4 and CD36.** n=3. * represents *p* < 0.05.
